# The Efficacy of Fat Grafting on Treating Post-Mastectomy Pain with and without Breast Reconstruction: A Systematic Review and Meta-Analysis

**DOI:** 10.3390/curroncol31040152

**Published:** 2024-04-04

**Authors:** Jeffrey Chen, Abdulrahman A. Alghamdi, Chi Yi Wong, Muna F. Alnaim, Gabriel Kuper, Jing Zhang

**Affiliations:** 1 Department of Medicine, Faculty of Health Sciences, McMaster University, Hamilton, ON L8S 4L8, Canada; chenj550@mcmaster.ca (J.C.); stephanie.wong@medportal.ca (C.Y.W.); 2 Division of Plastic Surgery, McMaster University, Hamilton, ON L8S 4L8, Canada; abdulrahman.alghamdi@medportal.ca; 3 College of Medicine, King Faisal University, Al Ahsa 31982, Saudi Arabia; 218009149@student.kfu.edu.sa; 4 Department of Medicine, University of British Columbia, Vancouver, BC V6T 1Z4, Canada; gnkuper@student.ubc.ca; 5 Division of Plastics and Reconstructive Surgery, Department of Surgery, The Ottawa Hospital, Ottawa, ON K1H 8L6, Canada

**Keywords:** post-mastectomy pain syndrome, mastectomy, patient-reported outcomes, surgical complication, fat grafting

## Abstract

Post-mastectomy pain syndrome (PMPS), characterized by persistent pain lasting at least three months following mastectomy, affects 20–50% of breast surgery patients, lacking effective treatment options. A review was conducted utilizing EMBASE, MEDLINE, and all evidence-based medicine reviews to evaluate the effect of fat grafting as a treatment option for PMPS from database inception to 29 April 2023 (PROSPERO ID: CRD42023422627). Nine studies and 812 patients in total were included in the review. The overall mean change in visual analog scale (VAS) was −3.6 in 285 patients following fat grafting and 0.5 in 147 control group patients. There was a significant reduction in VAS from baseline in the fat grafting group compared to the control group, *n* = 395, mean difference = −2.17 (95% CI, −2.95 to −1.39). This significant improvement was also noted in patients who underwent mastectomy without reconstruction. Common complications related to fat grafting include capsular contracture, seroma, hematoma, and infection. Surgeons should consider fat grafting as a treatment option for PMPS. However, future research is needed to substantiate this evidence and to identify timing, volume of fat grafting, and which patient cohort will benefit the most.

## 1. Introduction

Approximately 66% of patients with breast cancer, the most prevalent non-skin malignancy worldwide, require breast conserving surgery, while 32% undergo mastectomy [1,2]. Pain after mastectomy has long been considered a rare event related to damage to the intercostobrachial nerve [3]. However, post-mastectomy pain syndrome (PMPS), a chronic painful condition, affects around 20–50% of patients who undergo breast cancer surgery [4]. It is described as shooting and burning pain that manifests in the shoulder, axilla, arm, and chest wall that severely impacts quality of life, particularly through its debilitating impact on patients’ mental health, daily function, and financial stability [4,5,6]. Although the survival rate for breast cancer patients has significantly improved, especially in patients under 40 years old, the prevalence of PMPS remains high and potentially increases in breast cancer survivors [1]. With growing incidences of breast cancer throughout the population along with continuous advancements in survival, PMPS becomes increasing relevant, necessitating support for successful evidence-based analgesic methods.

Risk factors, such as pre-operative pain, axillary node dissection (ALND), anxiety, younger age, and radiation therapy, have been identified for PMPS. Notably, while ALND has been a standard component of breast cancer management, current clinical trials are exploring the possibility of safely de-escalating axillary surgery, in light of its association with worsened patient-reported outcomes [7]. However, there remains no standard treatment or recognized clinical guidelines for PMPS [8]. Botulinum toxin, neuromodulation, regional anesthesia, oral analgesia, and injection therapy have provided only temporary relief [9,10]. Autologous fat grafting, a procedure which involves the surgical transfer of adipose tissue from one area of the body to another, is a well-established technique in the field of reconstructive plastic surgery with uses such as for cosmesis and scar irritation [8]. It has been reported in multiple studies to improve the quality of life and alleviate pain in post-mastectomy pain syndrome patients [9,11,12]. While the mechanism through which autologous fat grafting helps reduce pain in patients after breast surgery still remains open to question, theories include loosening scar tissue contracture, decreasing inflammation, and more [8]. With an increased focus on the effect of fat grafting in recent years as a potential regimen for treating PMPS, there is a growing number of promising new evidence and research articles regarding this topic. While two previous systematic reviews have found a reduction in pain following fat grafting after mastectomy, these reviews may have limited power, as they included three and six studies, respectively [8,10]. Furthermore, neither review performed a meta-analysis nor examined complication rates associated with fat grafting. 

Improving the quality of life for breast cancer survivors experiencing PMPS is the cornerstone of this review. We hope to bridge a significant gap in the current literature by investigating the efficacy and safety as a potential treatment to alleviate the symptoms of PMPS. Therefore, the aim of this study was to perform a systematic review and meta-analysis on the current evidence on the efficacy of autologous fat grafting in the treatment of PMPS as well as its associated complications. Additionally, we performed a subgroup analysis of the pain-reducing effect of fat grafting on patients who have undergone mastectomy without reconstruction. The findings of this study may guide clinical decision making and shine a light on future research in PMPS treatment, ultimately improving quality of life of breast cancer survivors.

## 2. Materials and Methods

### 2.1. Search Strategy and Eligibility

This review was conducted according to the Preferred Reporting Items for Systematic Reviews and Meta-Analysis (PRISMA) guidelines and was registered on the International Prospective Register of Systematic Reviews (PROSPERO ID: CRD42023422627) [13]. One reviewer (JC) searched three online databases (Embase, MEDLINE, and EBM Review through OVID) for literature on fat grafting and post-mastectomy pain from database inception to 29 April 2023. Broad search terms included ‘Breast Neoplasms’, ‘Mastectomy’, ‘Fat Transfer’, ‘Fat Graft’, and similar phrases (Table 1). 

References of included studies were also screened using the same systematic approach. The research question and inclusion and exclusion criteria were established a priori. Inclusion criteria were as follows: (1) investigated fat grafting in patients with mastectomy with or without breast reconstruction; (2) conducted on human participants; (3) reported on clinical or patient-reported outcomes; (4) were of observational or experimental design; (5) contained original data; (6) papers published in English. The exclusion criteria were as follows: (1) reviews, conference abstracts, letters, case reports, and case series; (2) studies with outcomes that could not be appropriately isolated from patients or received breast-conserving surgery.

### 2.2. Screening

Systematic screening in accordance with PRISMA was performed in duplicate by four independent reviewers (SW, MA, JC, and GK) from title to full-text screening stages on Covidence (Melbourne, Australia). Conflicts were resolved by consensus or a third reviewer if consensus could not be reached. Agreement for title/abstract and full-text screening will be reported as a Kappa statistic interpreted according to Landis and Koch guidelines [14].

### 2.3. Data Extraction

Data extraction was completed in duplicate by two reviewers (SW and MA). Conflicts were resolved by consensus or a third reviewer if consensus could not be reached. The following variables were extracted: study and patient characteristics, surgery type/details, anesthesia details, previous radiation and radiotherapy, and chemotherapy, associated lymph node dissection, follow-up duration, average and maximum pain score, mean change in pain score, and conclusion. Authors derived means/standard deviations (SDs) when they were unavailable using the formulae proposed by Walter et al. and Wan et al., or by assuming a correlation coefficient of 0 [15,16]. The intervention group was defined as patients who received autologous fat grafting and the control group was defined as patients who did not.

### 2.4. Risk of Bias Assessment

The Newcastle Ottawa Scale and the Cochrane Collaboration RoB 2 tool were used to assess risk of bias in non-randomized and randomized studies, respectively [17]. Risk of bias was assessed in duplicate by two reviewers (SW and MA). Conflicts were resolved by consensus or a third reviewer if consensus could not be reached. 

### 2.5. Outcomes

The primary outcomes in this review were the difference in postoperative pain visual analogue scale (VAS) score between baseline and the latest follow-up. Outcomes were compared between breast surgery groups with and without fat grafting. Subgroup analysis was performed between patients who have underwent mastectomy and those who underwent breast reconstruction following mastectomy. 

### 2.6. Data Analysis

Descriptive statistics were used to summarize study and patient characteristics along with rates of complications. Meta-analysis was conducted using Review Manager [build 5.4.1] (Copenhagen, Denmark). For the primary outcome, the VAS score, mean difference (MD) with 95% confidence intervals based on the heterogeneity of measures was used across studies. The minimal clinically important difference (MCID) for VAS was identified in the literature review as 10 on the 100 mm scale [18].

A *p*-value of <0.05 was considered statistically significant. Subgroup analyses were conducted for mastectomy with and without breast reconstruction. Heterogeneity was determined using random versus fixed effects models. Sensitivity analysis was conducted using leave-one-out analysis.

## 3. Results

### 3.1. Study Selection

**Figure 1:**
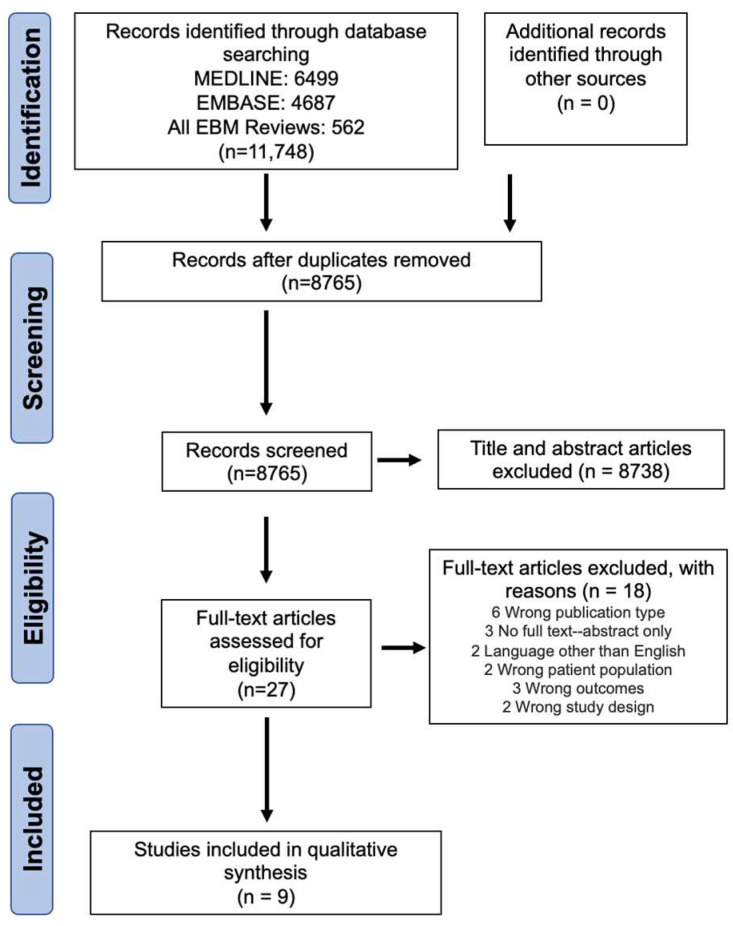
PRISMA flow diagram.

Initial search of online databases yielded 11,748 results, of which nine articles that examined pain associated with fat grafting following mastectomy were included in this review (Figure 1).

### 3.2. Screening Agreeability

The interrater reliability for title, abstract, and full-text screening was calculated utilizing Cohen’s kappa through Covidence [19]. The kappa result was interpreted as follows: 0.41 to 0.60 as moderate agreement, 0.61 to 0.80 as substantial, and 0.81 to 1.00 as almost perfect agreement [12]. The average kappa value was 0.49 for the title and abstract screening and 0.56 for the full-text screening, which corresponded to moderate interrater agreement. All disagreements were thoroughly discussed and resolved by consensus.

### 3.3. Study Characteristics

The characteristics of the nine included studies are summarized in Supplementary Table S1 [9,11,12,20,21,22,23,24,25]. These studies consisted of two randomized controlled trials (RCTs), six prospective studies, and one case–control study, published between 2009 and 2022. Collectively, these studies included a total of 812 patients who had undergone mastectomy. Among them, 453 patients received fat grafting post-mastectomy, while 359 did not receive fat transfer. The mean age of enrolled patients ranged from 41 to 61 years in the control group, and from 48 to 63.8 years in the intervention group. Follow-up durations ranged from 3 months to 1 year for prospective studies, 6 months in both RCTs, and 32.91–36.15 months in the single case–control study. The mean total amount of fat injected in the intervention group was 75 cc per breast.

Of the nine studies, five studies focused on patients who underwent only mastectomy, three studies included mastectomy with implant-based breast reconstruction, and one study included both implant-based and adipose tissue-based autologous reconstruction following mastectomy. All studies reported the use of radiotherapy. Of the 812 patients, 627 patients (84.8%) underwent radiotherapy prior to fat grafting. In the intervention group, 384 out of 453 (80.1%) patients received radiotherapy, compared to 243 out of 359 (67.7%) patients in the control group. Axillary lymph node dissection was reported in six studies, accounting for 467 patients. Among these, 343 patients (73.4%) underwent the axillary lymph node dissection—222 out of 303 (73.3%) in the intervention group and 121 out of 164 (73.8%) in the control group. Anesthesia type used during the fat grafting procedure was reported in 8 of the 9 studies, and involved 636 patients. For fat grafting, local anesthesia with sedation was utilized in five studies (297 patients), whereas the remaining three studies used general anesthesia (72 patients).

### 3.4. Risk of Bias Assessment

**Figure 2:**
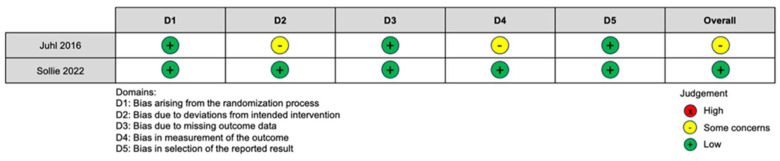
RCT risk of bias assessment [12,23].

Of the two RCTs assessed using the Cochrane RoB 2 tool, Juhl et al. had some concerns, mainly regarding measurement of outcome and deviations from intended interventions. Sollie et al. had a low overall risk of bias (Figure 2). There were six cohort studies and one case–control study assessed using the Newcastle Ottawa Scale. The scores ranged from 5 to 8 stars, with a median of 7 out of a maximum of 9 (Table 2). The worst domain category was comparability; four out of seven (57.1%) non-randomized studies scored 0 in this category. These studies did not control the potential confounders, such as age, BMI, tumor stage, requirement of chemotherapy, and type of reconstruction between the control and intervention groups. 

### 3.5. Post-Mastectomy Pain Syndrome

Among the nine included studies, VAS was the most popular pain scale used (n = 5), followed by BREAST-Q (n = 2) and Neuropathic Pain Symptom Inventory (NPSI) (n = 2). Numerical Rating Scale (NRS) and LENT-SOMA were each utilized by one study. All but one study (88.9%) individually reported statistical significance in the reduction of post-mastectomy breast pain in patients treated with fat tissue grafting [12]. 

### 3.6. VAS

**Figure 3:**
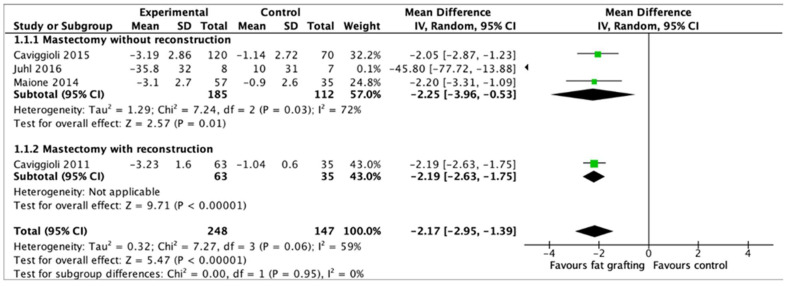
Forest plot of the effect of fat grafting on VAS score. IV, inverse variance; CI, confidence interval; df, degrees of freedom; I^2^, heterogeneity statistic; Z, z-statistic [11,20,23,24].

There were four studies (395 patients) eligible for meta-analysis, all of which compared patients who received fat grafting with patients who did not (Figure 3) [11,20,23,24]. Meta-analysis showed significant reduction in VAS score from baseline in the fat grafting group compared to the control group (MD: −2.17, 95% CI: [−2.95; −1.39], I^2^: 59%, *p* < 0.00001). This is a clinically significant reduction of 22 mm on a 100 mm scale [18]. Using the leave-one-out sensitivity analysis, the removal of the Juhl 2016 study resulted in a notable reduction in heterogeneity, accounting for a 59% change from a baseline of 0% [23]. 

For subgroup analysis, three studies included 185 patients who underwent fat grafting following mastectomy without reconstruction [20,23,24]. Fat grafting was shown to be associated with significantly decreased VAS compared to the control group (MD: −2.25, 95% CI: [−3.96; −0.53], I^2^: 72%, *p* = 0.01). Test for subgroup differences with mastectomy with reconstruction was not possible due to lack of studies.

Of the five studies (432 patients) which used the VAS scale, the mean baseline VAS scores (three studies, n = 242) were 7.1 and 6.7 for the fat grafting group (n = 165) and control group (n = 77), respectively. The mean VAS scores (three studies, n = 242) at the latest follow-up were 2.6 and 5.8 for the intervention (n = 165) and control (n = 77) groups, respectively. The overall mean change (five studies, n = 432) in VAS was −3.6 in 285 patients following fat grafting and 0.5 in 147 control group patients. 

### 3.7. NPSI

Two studies (50 patients) utilized the NPSI scale [12,23]. The mean total baseline NPSI scores were 19.1 and 19.2 for the fat grafting group and control group, respectively. In the intervention and control groups, respectively, the mean NPSI scores at the latest follow-up (6 months) were 12.6 and 16.0, resulting in a mean reduction of 6.5 and 3.2. One study which utilized both the NPSI and NRS rating scales did not find a significant analgesic effect associated with fat grafting in post-mastectomy patients.

### 3.8. Complications

The rates of complications in patients who underwent fat grafting are outlined in Table 3. A total of eight studies, involving 435 patients, reported on the incidence of complications after fat grafting. Complications were generally rare, with the most frequent being capsular contracture at 1.4%. This was followed by hematoma, seroma, and implant infection, each at 0.5%, and implant rupture at 0.2%. Notably, one study likely contributed most to heterogeneity, as it reported a revision surgery rate of 21.4% (18 out of 84 patients), though the specific reasons for these surgeries were not provided [21]. In the remaining seven studies, no revision surgeries were reported at the latest follow-up.

## 4. Discussion

This is the first meta-analysis evaluating the efficacy of autologous fat grafting for PMPS. There was a statistically significant decrease in VAS scores for patients treated with fat grafting compared to untreated patients. The substantial reduction in pain score was also found in the patient group that underwent mastectomy without reconstruction. The decrease in VAS scores was clinically important as well. Complications following fat grafting were rare and a qualitative decrease in NPSI scores was seen. This study demonstrates superior pain reduction through fat grafting for post-mastectomy patients. However, there is limited high-quality research and standardized outcomes on this topic, despite the prevalence of PMPS.

PMPS has significant psychosocial impact on patients; techniques and methods that can alleviate the extent of pain experienced can greatly improve patients’ quality of life [4,5]. We observed a significant reduction in long-term post-mastectomy VAS scores among fat-grafted patients when compared to patients who did not undergo treatment for PMPS [18]. There was a mean VAS reduction of 2.2 on a 10-point scale, greater than the MCID. The exact mechanism behind pain reduction is unclear but may involve adipose-derived stem cells and anti-inflammatory factors within the grafted fat, reducing neuropathic hypersensitivity and neuroinflammation [26,27]. Currently, the gold standard for PMPS measurement is patient-reported scales, which may have potential limitations and biases due to the subjective nature of patient-reported outcome measures [28]. Of which, VAS, a validated scale, has shown sensitivity to treatment effects and correlation with other pain assessment tools [5]. While VAS is particularly strong at measuring differences in pain intensity between two time points, and the tool itself is straightforward in its administration, its disadvantages include susceptibility to misinterpretation by specific populations, ambiguity of end points, and its subjective nature [5,28]. Like VAS, the NPSI, a well-validated scale for neuropathic pain, faces challenges when pooling results statistically on a larger scale [5,29]. To provide more accurate results for investigations into pain modality treatments, objective measures such as pain biomarkers and neurological imaging are currently being explored, which would enable access to a greater and alternate view into the management of pain [28]. 

Fat grafting in the breast has been proved to be a well-tolerated procedure [30,31]. Rates of major complications, such as hematoma, infection, and seroma, range from 2 to 3.6%, and are predominantly seen in cases where fat grafting was accompanied by reconstruction or revision. Specifically, our review found a lower rate of common surgical complications following fat grafting than the rates mentioned above, potentially because most patients in this review underwent fat grafting as a standalone procedure. It is important to note that our review did not account for any additional procedures performed in conjunction with fat grafting that could contribute to surgical site complications [30]. One study recruited in this review, by Calabrese et al., likely contributes to heterogeneity due to its high rate of revision surgery [21]. Nonetheless, Calabrese et al. reported a significantly lower rate of complications in the fat grafting patient group compared to its control group [21]. Overall, while our analyses and results demonstrate optimism for reduced complications and increased safety in autologous fat grafting in PMPS, the limited sample and study size of our review along with the presence of marked heterogeneity suggest that further research is needed.

Despite increased breast cancer survival rates and the vast number of breast surgeries today, treatment for chronic post-breast surgery pain remains as a significant challenge due to the lack of sufficient clear or well supported management guidelines [8]. A recent study has underscored the benefits of prepectoral immediate breast reconstruction. This method has been identified as both safe and cost-effective, while offering patients similar quality of life and cosmetic results compared to the traditional submuscular approach, thereby spotlighting the potential of surgical techniques to enhance post-mastectomy quality of life [32]. Our findings reveal that fat grafting post-breast surgery may offer substantial long-term pain relief without an increased risk of complications. This is particularly important for patients and health care providers, suggesting fat grafting’s potential to help those battling chronic pain. However, additional studies with standardized and objective outcome measurement are essential to validate effects of this treatment. Ultimately, the role of fat grafting in the multimodal approach for treating PMPS alongside physical and cognitive therapy, nerve blocks, medications, and surgical interventions shows promise and should continue to be investigated.

### Limitations

This review has several limitations. First, it includes only English-language studies, potentially introducing a language bias. Second, grey literature was not searched, which could skew results toward studies that favor fat grafting. Third, there was notable heterogeneity in the reporting of outcomes across the included studies, affecting the reliability of pooled effect estimates. To mitigate this, we pooled the latest reported VAS scores, which ranged from 3 months to 1 year post-operation. Lastly, many studies were ineligible for meta-analysis due to the use of multiple pain scales or the lack of a control group.

## 5. Conclusions

Autologous fat grafting following breast surgery may significantly reduce chronic pain compared to a control group and does not increase the risk and rates of complications. Future research is needed to further substantiate this evidence and to identify specific patient demographics who will benefit the most from lipo-transfer, such as those undergoing radiotherapy or lymph node dissection, type of reconstruction, as well as to determine the timing of fat grafting and volume of grafting that will be the most efficacious in alleviating PMPS.

## Figures and Tables

**Table 1:** Search criteria on Medline, EMBASE, and EBM reviews (through OVID).

Query	Results from 29 April 2023		
	Medline	EMBASE	EBM
1. Breast Neoplasms/or breast surgery.mp.	330,797	44,072	19,463
2. mastectomy.mp. or Mastectomy/	46,860	76,214	6068
3. breast reconstruction.mp. or Mammaplasty/	19,265	23,371	1056
4. lumpectomy.mp. or Mastectomy, Segmental/	12,004	23,079	1300
5. postmastectomy.mp.	2504	3393	389
6. post-mastectomy.mp.	1429	2787	411
7. post-mastectomy pain syndrome.mp.	54	108	44
8. postmastectomy pain syndrome.mp.	74	104	33
9. fat.mp.	325,443	493,736	41,334
10. fat transfer.mp.	659	795	46
11. fat transplantation.mp.	510	654	35
12. fat graft.mp.	1675	2143	106
13. fat grafting.mp.	2591	2955	127
14. Tissue Expansion/	2476	3916	53
15. 1 or 2 or 3 or 4 or 5 or 6 or 7 or 8	349,324	123,617	23,147
16. 9 or 10 or 11 or 12 or 13 or 14	327,807	497,180	41,385
17. 15 and 16	6499	4687	562

**Table 2:** Non-randomized studies’ risk of bias assessment.

**Cohort Studies**			
**Study**	**Selection**	**Comparability**	**Outcome**
Caviggioli 2011 [11]	****		***
Caviggioli 2015 [20]	****		***
Cogliandro 2017 [22]	****	*	***
Lisa 2020 [9]	****		***
Maione 2014 [24]	****	*	***
Panettiere 2009 [25]	***		**
**Case–Control Studies**			
**Study**	**Selection**	**Comparability**	**Exposure**
Calabrese 2019 [21]	****	*	*

The Newcastle-Ottawa Scale is scored by awarding a point (*) for answers in 3 main categories. Possible total points are 4 (****) points for Selection, 2 (**) points for Comparability, and 3 (***) points for Outcomes.

**Table 3:** Complications associated with fat grafting.

Complications	Overall Rate in Intervention Group
Revision surgery	4.1% (n = 18/435)
Capsular contracture	1.4% (n = 6/435)
Hematoma	0.5% (n = 2/435)
Seroma	0.5% (n = 2/435)
Implant infection/dehiscence	0.5% (n = 2/435)
Implant rupture	0.2% (n = 1/435)
Implant exposure	0% (n = 0/435)

## Data Availability

No new data were created or analyzed in this study. Data sharing is not applicable to this article.

## Supplementary Materials

The following supporting information can be downloaded at: https://www.mdpi.com/article/10.3390/curroncol31040152/s1, Table S1. Study characteristics of included studies.
